# Translational genomics in cancer research: converting profiles into personalized cancer medicine

**DOI:** 10.7497/j.issn.2095-3941.2013.04.005

**Published:** 2013-12

**Authors:** Lalit Patel, Brittany Parker, Da Yang, Wei Zhang

**Affiliations:** 1 Department of Pathology, The University of Texas MD Anderson Cancer Center, Houston, TX 77030, USA;; 2 The University of Texas Graduate School of Biomedical Sciences, Houston, TX 77030, USA

**Keywords:** Cancer, genomics, translation, personalized medicine

## Abstract

Cancer genomics is a rapidly growing discipline in which the genetic molecular basis of malignancy is studied at the scale of whole genomes. While the discipline has been successful with respect to identifying specific oncogenes and tumor suppressors involved in oncogenesis, it is also challenging our approach to managing patients suffering from this deadly disease. Specifically cancer genomics is driving clinical oncology to take a more molecular approach to diagnosis, prognostication, and treatment selection. We review here recent work undertaken in cancer genomics with an emphasis on translation of genomic findings. Finally, we discuss scientific challenges and research opportunities emerging from findings derived through analysis of tumors with high-depth sequencing.

## Introduction

Sun Tzu stated in The Art of War, “If you know the enemy and know yourself, you need not fear the result of a hundred battles. If you know yourself but not the enemy, for every victory gained you will also suffer a defeat. If you know neither the enemy nor yourself, you will succumb in every battle.”

These words have held true with respect to the efforts of medical science to conquer cancer as a cause of death and suffering. There have been both occasions when the drivers of malignancy eluded curative efforts and also occasions when our diagnostic and therapeutic strategies have not met the task despite the underlying molecular biology of disease becoming more evident. Translational cancer research has accordingly benefited from both advances in our understanding of the enemy that cancer continues to be and the ongoing effort to evaluate and make better the suite of diagnostics, therapeutics, and rational decision making that underlie cancer treatment.

More than a decade into the post-genomic era, we have come to appreciate human malignancy as a condition derived from somatic aberrations in the human genome. Early studies enabled by oligonucleotide hybridization arrays proved to be highly informative, demonstrating a role for somatic copy number variations (CNVs)^1^, mutations^2^, and differential transcript expression^3^ as cancer promoting events. Current efforts build from these successes while benefiting from the rapid evolution of high throughput sequencing and bioinformatics techniques^4^. To this end, several coordinated multi-center efforts including The Cancer Genome Atlas (TCGA)^5^ and the International Cancer Genome Consortium (ICGC)^6^ have been organized to interrogate the genomes of dozens of cancer types. Several cancer genome sequencing studies have also been reported by independent groups^7^^-^^11^. Reviewed here are emerging themes from these studies and their applications to both the biology of cancer and new concepts in patient management.

## Molecular subtyping through integrative analysis

The cost of microarrays and high-throughput sequencing lends itself to the development of multiple molecular profiles per cancer type. For instance, gene expression, somatic mutation calls, and DNA copy number can each be assessed in a sample matched manner on large cohorts of clinical specimens. When such profiles are coupled with drug response and clinical outcomes annotation, integrative analysis can be performed to reveal clinically relevant molecular subsets. Early efforts demonstrated the value of genomic data integration using the NCI60 panel of cell lines to predict therapeutic response^12^^,^^13^. Recent work by the Cancer Cell Line Encyclopedia^14^ and Genomics of Drug Sensitivity in Cancer^15^ expanded this effort to include a larger panel of cell lines with more thorough genomic profiling, and has provided a suite of molecular diagnostics that may help better match patients to targeted therapies to which they respond.

Although efforts utilizing cell lines have proven to be informative, the most informative analysis would be of profiles generated from clinical cases. Genomic and clinicopathologic profiles made public by the TCGA provide a unique opportunity to decipher the molecular basis of the heterogeneity in clinical course taken by single diseases and in some cases reveal unexpected associations in molecular etiology across diseases. For instance, analysis by the TCGA of high grade serous ovarian cancer (OvCa) identified four distinct gene expression clusters: differentiated, immunoreactive, proliferative, and mesenchymal^16^. The same study also identified microRNA expression clusters C1, C2, and C3 of which microRNA cluster C1 associates with cases bearing a proliferative gene expression profile and C2 associates with messenchymal cases. Further interrogation by the TCGA determined that the C1 microRNA signature predicts diminished survival. Together these data suggest that microRNA networks define a significant regulatory mechanism and may distinguish actionable subtypes of clinical cases.

Pursuant to these findings, Yang *et al.*^17^ developed a computational pipeline (Master mIRna Analysis for Cancer moLecular subtype, MIRACLE) which aims to delineate the driver events and applied them to identify driver miRNAs for the mesenchymal signature of ovarian cancer. Using genes in the regulatory network, the study further characterized an integrated mesenchymal subtype significantly associated with poor survival in 459 serous OvCa cases from TCGA and 560 cases from three independent OvCa patient cohorts. The miRNA-regulatory network derived from this analysis consists of eight key miRNAs predicted to regulate 89% of the targets. Among them are not only well-established EMT inhibitors such as miR-200 family but also previously uncharacterized drivers such as miR-506 which Yang *et al.*^17^ demonstrated to be a novel EMT inhibitor by targeting SNAI2. Specifically, transfection of miR-506 augmented E-cadherin expression, inhibited cell migration and invasion, and prevented TGFβ-induced EMT, while force expression of SNAI2 abolished miR-506’s effect. In human samples, miR-506 expression correlated with decreased SNAI2, elevated E-cadherin, and beneficial prognosis. Exploring the therapeutic efficacy of miR-506 in OvCa, the study also demonstrated the suppression of EMT and tumor growth *in vivo* subsequent to treatment with nanoparticle-incorporated miR-506 in orthotopic OvCa mouse models. Integrative genomic analysis in this study has thus nominated miR-506 as both a prognostic marker and potential therapeutic indicator.

Similarly, the application Mutually Exclusive Modules in Cancer (MEMo), an integrative analysis pipeline leveraging correlation analysis and graph theory, was deployed using data from the TCGA to characterize networks in glioblastoma multiforme (GBM). In doing so, two regulatory networks were identified by a total of six genes, distilling out a small subset of putative drivers from hundreds of genetic events^18^. The advantage of this analysis is not only that it limits the number of genes for functional follow-up studies probing the biology of GBM, but also that it nominates a workable six-gene panel for evaluation as a putative clinical diagnostic.

Other integrative tools have also been developed and applied for this purpose. The ARACNe algorithm recently uncovered regulatory interactions driving epithelial-to-mesenchymal transformation using the same GBM data as the MEMo study^19^. Likewise, PARADIGM, a pipeline that integrates genomic profiles into a model of transcriptional and cell-signaling interactions, inferred activation of the FOXM1 signaling as a highly recurrent high-grade serous OvCa^16^. While small-molecule inhibitors to transcription factors remain a challenge to develop, the capacity to identify cancers driven by specific transcription regulators may prove beneficial when identifying what subset of patients to treat with these drugs as they become available.

The inevitable consequence of generating enough data to observe the natural subsets occurring between samples of the same cancer is that efforts to treat cancer will necessarily evolve to be more subsets-specific. For instance, differentiated OvCa is likely to require a different therapeutic strategy from that of cases with a molecular signature that is more proliferative. Getting to the point where we know what strategy is best for each molecular subtype will require the same level of focused investigation within each molecular subtype as the studies that have led to their identification. Thus, a theme of molecular subtyping compelling more personalized treatment plans and more precise contexts for therapy development has emerged from cancer genome studies.

## BRCA-driven ovarian cancer: a case of genomics driving personalization

In the general population, an estimated 1 in 300 to 800 individuals carries a BRCA1 or BRCA2 mutation^20^. And 8%-13% of women diagnosed with epithelial OvCa have a germline BRCA1 or BRCA2 mutation^21^^-^^23^. The mutation frequencies of BRCA1/2 raise to 16%-21% in serous subtype of ovarian cancer, which accounts for 70% of OvCa^21^^,^^23^^,^^24^. The risk of developing OvCa by age 70 years is 40%-50% for BRCA1 mutation carriers and 10%-20% for BRCA2 mutation carriers^25^^,^^26^. BRCA1 and BRCA2 mutations can be also found in primary fallopian tube and peritoneal cancers^27^.

Accumulating evidence^28^^-^^31^ shows that BRCA1/2 mutation-related OvCa cases have a discernibly diminished prognosis and platinum response rate compared to non-BRCA1/2 mutant OvCa cases. In a recent report, Yang and colleagues performed integrated analyses of multidimensional genomic and clinical data from 316 high-grade serous OvCa patients in TCGA project and observed that patients with BRCA1 and BRCA2 mutations had unequal clinical features^32^. Specifically, patients with BRCA1 mutations were younger at diagnosis and the 5-year survival rate of BRCA2 mutation carriers was significantly higher than that of wild-type cases. Among BRCA2 mutation carriers, 100% were sensitive to primary platinum chemotherapy compared with 80% of BRCA1-mutated and 85% of wild-type cases.

Similarly, patients with BRCA2 mutations had a longer platinum-free survival interval than did BRCA1-mutant and wild-type patients. The availability of genomic data profiling somatic mutations, DNA copy number alterations, and methylation in the TCGA for all the analyzed OvCa cases allowed the authors to evaluate molecular correlates in a quantitative manner. This analysis revealed that BRCA2 cases exhibited a more pronounced “mutator phenotype”, as defined by the number of total mutations across the whole exome whereas BRCA1 mutated cancers exhibited no significant enrichment of mutations. Subsequent to this report, two independent studies also provided supporting evidence that BRCA2 mutation is associated with a better prognosis in OvCa^33^^,^^34^, including a pooled observational study including 3,739 epithelial OvCa cases (909 BRCA1, 304 BRCA2 mutation carriers and 2,666 non-carriers), by Bolton *et al.*^33^ reporting that BRCA2 mutation carriers had the best prognosis.

Since BRCA2 mutations are associated with longer platinum-free survival durations than are BRCA1 mutations and BRCA wild-type, a patient’s BRCA status may influence the choice of agents for adjuvant chemotherapy. Recent findings^35^^,^^36^ demonstrate that PARPi have cytotoxic effects on BRCA1- or BRCA2-deficient cells. The prevailing explanation for these findings center on a phenomenon called synthetic lethality^37^. Promising results from multiple clinical trials in BRCA-associated carcinomas, including OvCa, have been reported^38^^-^^41^.

One important consideration is whether differentials in response to platinum-based chemotherapy between BRCA1- and BRCA2-mutated ovarian cancers observed in recent studies may also be true with respect to the therapeutic response elicited by PARP inhibitors. Early clinical trials of PARP inhibitors, although statistically underpowered at their current sample size to detect differences in efficacy between the BRCA gene mutations, demonstrate notable trends. A study by Gelmon *et al.*^41^ included 11 BRCA1 and 5 BRCA2 mutated OvCa patients treated by PARPi and showed a 60% (3 of 5) response rate for BRCA2-mutant versus 24% (11of 60) for BRCA-wild-type and 36% (4 of 11) for BRCA1-mutant cases. A similar trend was shown in the cohort that received 400 mg of olaparib twice daily^39^. These marginal, but promising results indicate that further stratification based on BRCA1 and BRCA2 mutation status may be needed to evaluate the differential effects of PARPi treatment in individuals. In addition, upcoming trials of PARP inhibitors in ovarian cancer that specifically enrich for BRCA1 and BRCA2 carriers may be at particular risk for confounding biases in treatment response if differences in between these two biologically distinct groups are not considered.

## Oncogenic gene fusions: a class of tumor defining genomic events

Originally associated with blood leukemias, fusion genes have become an emerging class of oncogenes in solid tumors. Fusion genes are two previously separate genes that rearrange forming a novel “hybrid” gene, containing both of the original genes. The first discovered and most widely characterized fusion gene, BCR-ABL1, occurs in 95% of chronic myeloid leukemia patients^42^. Since then, with the advent and commercial availability of next-generation sequencing, more fusions began to be discovered in solid tumors^43^. Next-generation sequencing allowed research groups to perform sequencing reactions rapidly and at a lower cost than previous reactions did. This greatly pushed efforts to sequencing a greater variety of tumor types, and thus lead to the identification and characterization of more fusions. These efforts collectively lead to development of drug inhibitors which have showed vast therapeutic benefit.

Fusion genes can form via translocations in which chromosomes exchange the location of entire chromosome arms, deletions in which a segment of DNA is deleted between two consecutive genes, inversions in which a segment of DNA is inverted bringing two distant genes into the same open reading frame, or tandem duplications in which two genes in a region of microhomology are amplified and tiled next to one another. The TMPRSS2-ERG fusion is an example of a fusion forming via deletion, which results in the ERG gene put under the control of the androgen-regulated promoter *TMPRSS2.* This results in overexpression of the ERG oncogene leading to tumorigenesis^44^. The *FGFR3-TACC3* fusion gene found in GBM, bladder, and lung cancers, is an example of a fusion forming via tandem duplication. Both genes are amplified and tiled next to one another, leading to both genes occurring in the opposite direction as before the fusion event^45^. The BCR-ABL1 fusion is an example of translocation, in this case specifically between chromosomes 9 and 22^42^.

Fusion genes are attractive as diagnostic tools and therapeutic targets. The first fusion gene to be targeted was *BCR-ABL1*, where the tyrosine kinase inhibitor, imatinib, targeted the constitutively activated ABL1 kinase, and was approved for use by the Food and Drug administration in 2001. Another targeted fusion, the *PML-RARA* fusion, which occurs in 95% of acute promyelocytic leukemia patients found vast therapeutic benefit when treated with drug tretinoin^46^. Futhermore, the FGFR family fusions, which recently have been discovered in a variety of cancers including breast^47^, lung^47^, GBM^48^, and bladder cancers^49^, are uniquely targetable due to overexpression of the tyrosine kinase FGFR. Future efforts are involved with discovering means to target these fusion genes in diverse cancers.

Fusion genes are oncogenic via a variety of different mechanisms, including constitutive activation or overexpression of an oncogene. As mentioned previously, the *BCR-ABL1* oncogene forms via reciprocal translocation and encodes a constitutive activated tyrosine kinase, ABL1. The addition of *BCR* to the *ABL1* gene allows for receptor dimerization and therefore constitutive activation, where the receptor is maintained within the cytoplasm where its signals continually propagates downstream signaling cascades^50^^,^^51^. Similarly, the *FGFR3-TACC3* fusion gene has been proposed to exert its oncogenic phenotype via constitutive dimerization^45^^,^^48^^,^^49^. Specifically, the tacc3 protein contains a coiled-coil domain in the C-terminal that is retained upon formation of the *FGFR3-TACC3* fusion. This coiled-coil domain is hypothesized to allow constitutive dimerization of the fusion, which then maintains activity even in the absence of ligand^47^. This can then lead to constitutive activation of known downstream oncogenes, such as ERK and STAT3^45^^,^^49^. Interestingly, other dimerization domains have been described in a variety of fusion genes, all which contain FGFR family members^47^. Exactly how these dimerization domains allow oncogenic FGFR signaling remains to be elucidated.

Another way that oncogenic fusions can be overexpressed is via loss of microRNA regulation. MicroRNAs (miRNAs) are small, endogenous RNA molecules that can lead to mRNA degradation or can inhibit translation. The miRNAs regulate specific mRNA when their seed sequence matches one within the 3’ untranslated region (UTR) of a specific mRNA. Each miRNA has the potential to regulate hundreds of different mRNAs. The *FGFR3-*TACC3 fusion gene is one which can bypass microRNA regulation, via loss of the 3’ untranslated region on FGFR3. Specifically, upon formation of the fusion the 3’ UTR of FGFR3 lost. This 3’ UTR is under tight control of the microRNA 99a (miR-99a), which is very high in normal brain and in GBM. This explains why there is little wild-type FGFR3 found in both normal brain and GBM. However, upon formation of the *FGFR3-TACC3* fusion, this mRNA is then able to bypass signaling and is overexpressed^45^. A similar mechanism is observed with the *MYB-NFIB* fusion in adenoid cystic carcinoma of the head and neck, which occurs via translocation of chromosomes 6 and 9. The *MYB* gene encodes the oncogenic Myb transcription factor, which is overexpressed in a variety of cancers. The 3’ UTR of *MYB* is lost upon formation of the fusion, where it can then bypass microRNA signaling^52^.

Yet another mechanism by which fusion genes can exert their oncogenic phenotype occurs when an oncogene comes under the control of another genes’ more potent promoter. An example of this is the *TMPRSS2-ERG* fusion gene in prostate cancer. A segment between both genes is deleted which results in the *ERG* oncogene being in control of the *TMPRSS2* promoter. This promoter is androgen regulated, to where under normal conditions *TMPRSS2* is only expressed in prostate tissues when androgen is available. However, upon formation of the fusion, the *ERG* gene is therefore under control of this promoter, leading to the overexpression of ERG when androgen is present^44^. Similarly, another fusion gene found in prostate cancer links the SLC45A3 fusion to the same Ets family of transcription factors, although the prevalence is lower than *TMPRSS2-ERG* fusions^53^. A similar mechanism has recently been described linking the *SLC45A3* gene to *FGFR2*, where the *FGFR2* receptor tyrosine kinase is now under the control of the androgen regulated *SLC45A3*^47^. It is possible that TMPRSS2-fusion positive prostate cancer patients would uniquely responsive to androgen deprivation therapy, as this would limit the amount of androgen-induced oncogene being expressed. Given that many of these fusions are with genes that are members of the ETS-family, fusion-positive cases may also be uniquely served by inhibitors developed against this family of transcription factors. Patients with *SLC45A3-FGFR2* fusions may also benefit from FGFR inhibitor therapy to combat oncogenic signaling conferred by *FGFR2* activity.

Future efforts towards targeted cancer therapy should include developing drugs with the potential to inhibit the gene-products of oncogenic fusions. However, given the fusion-specific nature of tumor-biology in lesions driven by gene-fusions, implementation of such treatments would be most effective when treating patients of known gene fusion status. In other words, drugging gene-fusions being an exercise in targeting individual cancers on the basis of patient-specific somatic events makes this class of targets naturally suited for personalized medicine.

## Future directions and challenges: intra-tumoral heterogeneity and resistance

While the promise of more targeted precision therapy is hopeful, observations from clinical trails of targeted therapy demonstrate heterogeneity in treatment response even among lesions where drivers are known^54^^-^^57^. Innate and acquired resistance to targeted therapy accordingly presents a formidable challenge to translational efforts aimed at converting genomic findings into effective therapy. This has led some to parameterize treatment response using principles from evolutionary biology^58^. Specifically this view is predicated on the notion that tumors are heterogeneous populations of cancer cells that evolve through clonal and subclonal expansion to dynamically repopulate lesions under the selective pressure of systemic therapy. If this is true, then we may find the keys to unlocking durable treatment responses in the evolutionary behavior of tumors.

Only recently have genomic techniques capable of resolving intratumoral heterogeneity become available. Recent high-depth whole genome sequencing of lung cancers revealed the bi-clonal composition of tumors in both a smoker and a never smoker^59^, lending support to notion that solid tumors can be heterogeneous. Similar high-depth sequencing of eight paired primary and replaced acute myeloid leukemia cases demonstrated that resistance to chemotherapy emerged, at least in this subset of cases representing a hematologic malignancy, through the expansion and evolution of subclones present in the primary setting^60^.

Further advances in sequencing coupled with what we’re learning from early tumor heterogeneity studies may help with designing rational regimens and combinations of treatment to overcome resistance and relapse. However, as we’ve learned from the genomic profiling across tumor cohorts, data in its pure form is not sufficient to address unmet needs. Instead it is the combination of well designed data collection with creative analytical approaches that lead to new and informative insights. Returning to the wisdom of Sun Tzu, since we have known that cancer is an enemy that uses genome editing to perpetually evolve, our pursuit of durable and curative therapeutic responses will require our treatment strategies to evolve more rapidly than our adversary. One strategy would be to slow tumor evolution down, an area of cancer biology we do not sufficiently understand at present to properly exploit and therefore need to study further. Another would be to become more dynamic therapists whose treatment plans for individual patients evolve to keep pace with the moving target individual lesions are showing themselves to be.
